# Supplementary material for: TGFβ2-induced formation of lipid droplets supports acidosis-driven EMT and the metastatic spreading of cancer cells
Source: Nat Commun. 2020 Jan 23;11:454. doi: 10.1038/s41467-019-14262-3 (PMC6978517; doi:10.1038/s41467-019-14262-3)
Supplement: Supplementary file 1 — Supplementary Information [file 41467_2019_14262_MOESM1_ESM.pdf]

Supplementary Information

TGFβ2-induced formation of lipid droplets supports acidosis-driven EMT and the metastatic spreading of cancer cells

Corbet et al.

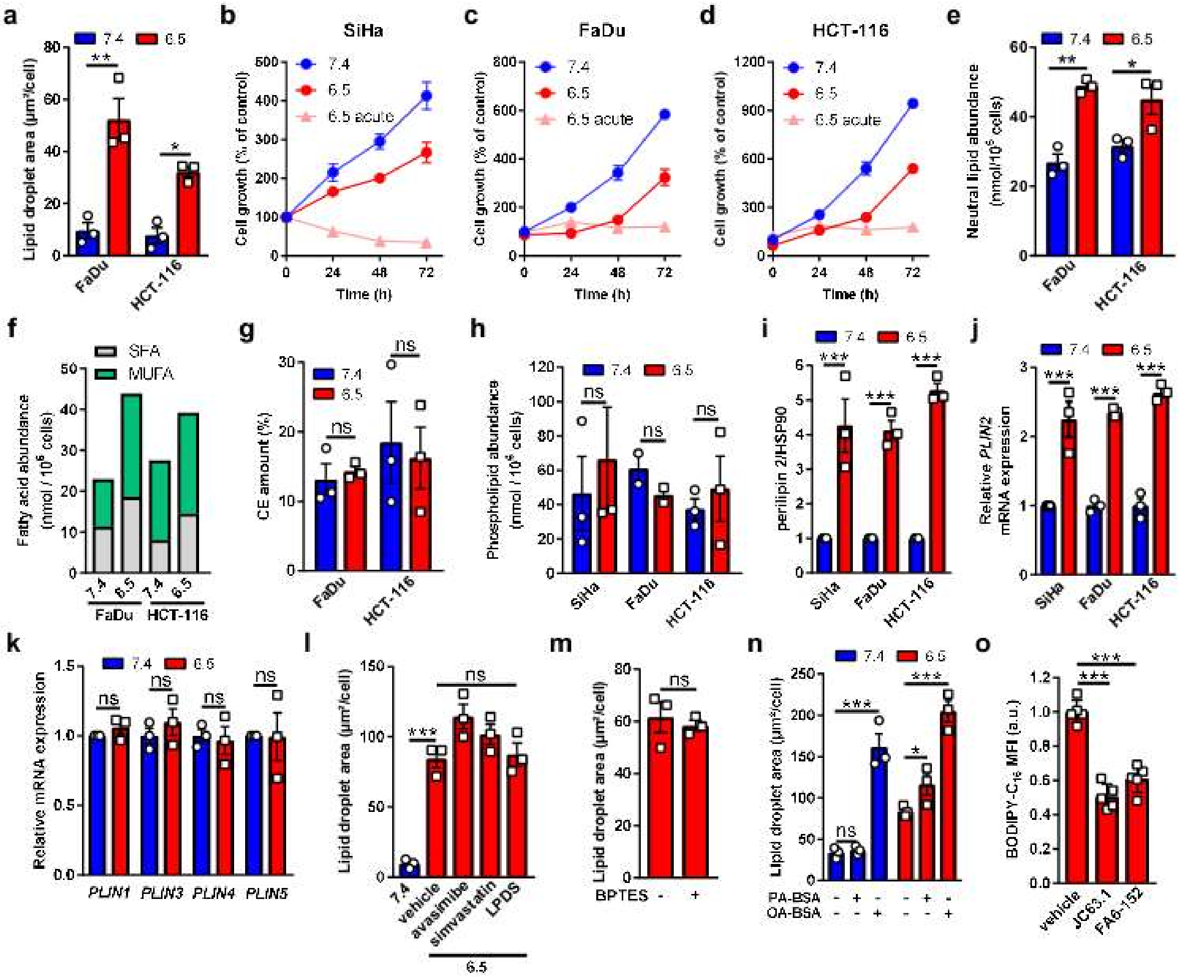

Supplementary Figure 1: Acidosis-adapted cancer cells exhibit accumulation of lipid droplets in a CD36- and DGAT1-dependent manner. (**a**) LD area in native and acidosis-adapted cancer cells. (**b-d**) Time courses of the growth of native, chronically and acutely pH6.5-exposed SiHa (**b**), FaDu (**c**), and HCT-116 (**d**) cancer cells. (**e**) Neutral lipid abundance in native and acidosis-adapted cancer cells. (**f-g**) Abundance of SFA and MUFA (**f**) and cholesteryl esters (**g**) in the neutral lipid fraction of native and acidosis-adapted cancer cells. (**h**) Phospholipid abundance in native and acidosis-adapted cancer cells. (**i-j**) quantification of protein expression (**i**) and mRNA expression (**j**) for *PLIN2* in native and acidosis-adapted cancer cells. (**k**) mRNA expression for other *PLIN* genes in native and acidosis-adapted SiHa cells. (**l**) LD content in native and acidosis-adapted SiHa cells following treatment with 5 µM avasimibe, 1 µM simvastatin or upon incubation in a medium containing lipoprotein-deficient serum (LPDS). (**m**) LD content in forskolin-treated acidosis-adapted SiHa cells incubated for 24h in a full medium with or without 20 µM BPTES. (**n**) LD content in native and acidosis-adapted SiHa cancer cells following treatment with 100 µM BSA-conjugated palmitate (PA-BSA) or oleate (OA-BSA) for 24h. (**o**) Quantification for BODIPY FL C_{16} uptake in acidosis-adapted SiHa

cells after incubation with the indicated CD36 blocking antibodies. Data are represented as mean ± SEM of three independent experiments (with ≥6 technical replicates). Significance was determined by Student's t-test (**m**), one-way ANOVA (**l, o**), or two-way ANOVA **(a, e, g-k, n**) with Bonferroni multiple-comparison analysis. *p<0.05; **p<0.01; ***p<0.001; ns, not significant. Source data are provided as a Source Data file.

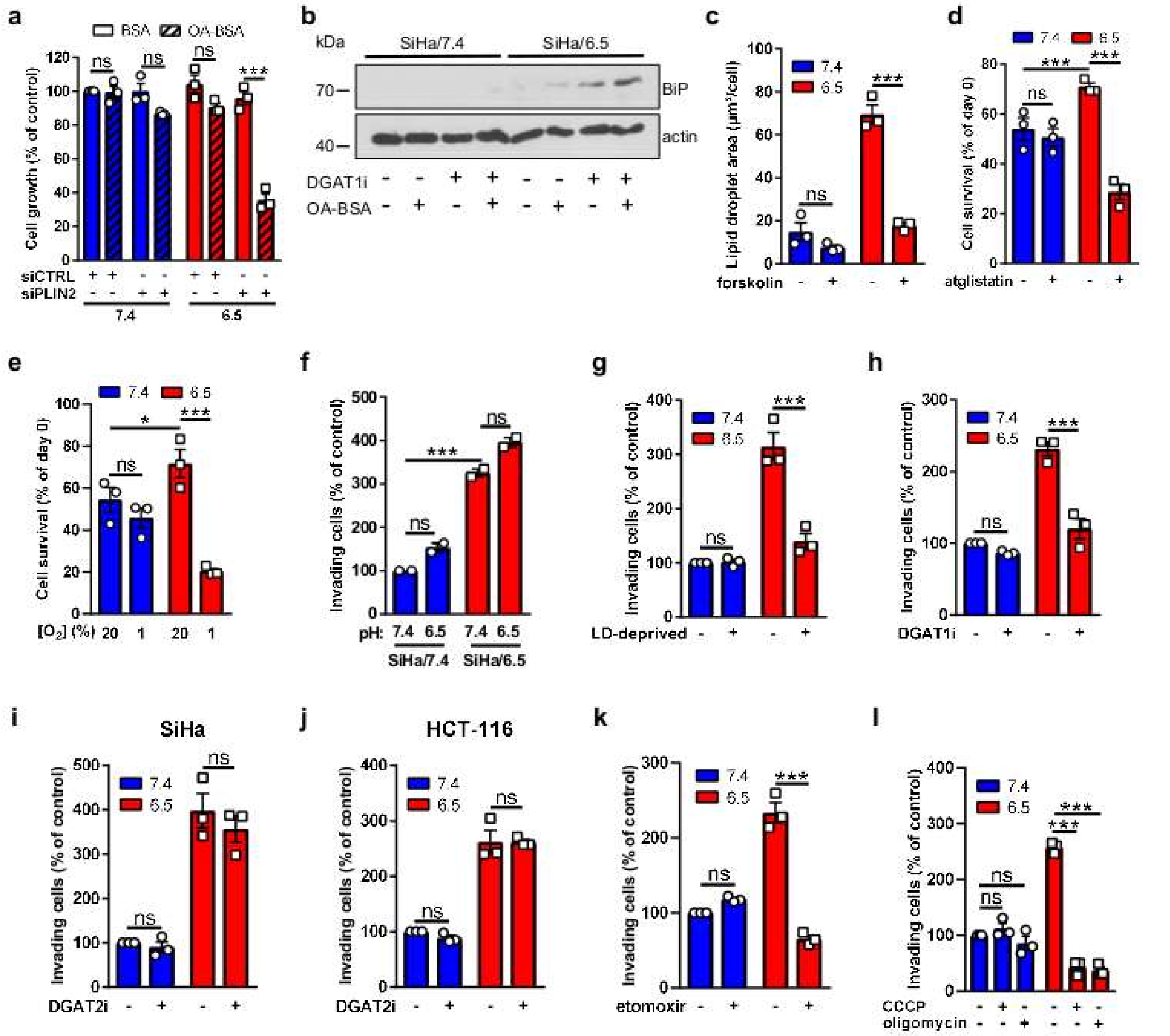

Supplementary Figure 2: Lipid droplets support the survival and invasiveness of acidosis-adapted cancer cells. (**a**) Cell growth extent for native and acidosis-adapted HCT-116 cells following transfection of control or PLIN2-targeting siRNA sequences and treatment with 50 µM BSA-conjugated oleic acid (OA) for 72h. (**b**) Representative immunoblotting for BiP (ER stress marker) in native and acidosis-adapted SiHa cells following treatment with 15 µM A922500 (DGAT1i) and/or 50 µM BSA-conjugated oleic acid (OA) for 48h. (**c**) LD content in native and acidosis-adapted HCT-116 cells following treatment with 10 µM forskolin for 24h. (**d-e**) Survival capacity of native and acidosis-adapted HCT-116 cells for 3 days in 0.1% serum-containing medium following treatment with 10 µM atglistatin (**d**) or in a dialyzed serum-containing medium without glucose nor glutamine under normoxic (20% O_2) or hypoxic (1% O_2) conditions (**e**). (**f**) Invasion capacity of native and acidosis-adapted SiHa cells placed at the indicated pH during the assay. (**g-h**) Invasion capacity of native and acidosis-adapted HCT-116

cells following treatment for 24h with 10 µM forskolin (**g**) or with 15 µM A922500 (DGAT1i) (**h**). (**i-j**) Invasion capacity of native and acidosis-adapted SiHa cells (**i**) and HCT-116 (**j**) following treatment for 24h with 10 µM PF-06424439 (DGAT2i). (**k-l**) Invasion capacity of native and acidosis-adapted HCT-116 cells following treatment with 30 µM etomoxir (**k)** or 100 µM CCCP and 1 µg/ml oligomycin (**l**). Data are represented as mean ± SEM of three independent experiments (with ≥6 technical replicates). Significance was determined by two-way ANOVA (**a, c-l**) with Bonferroni multiple-comparison analysis. *p<0.05; ***p<0.001; ns, not significant. Source data are provided as a Source Data file.

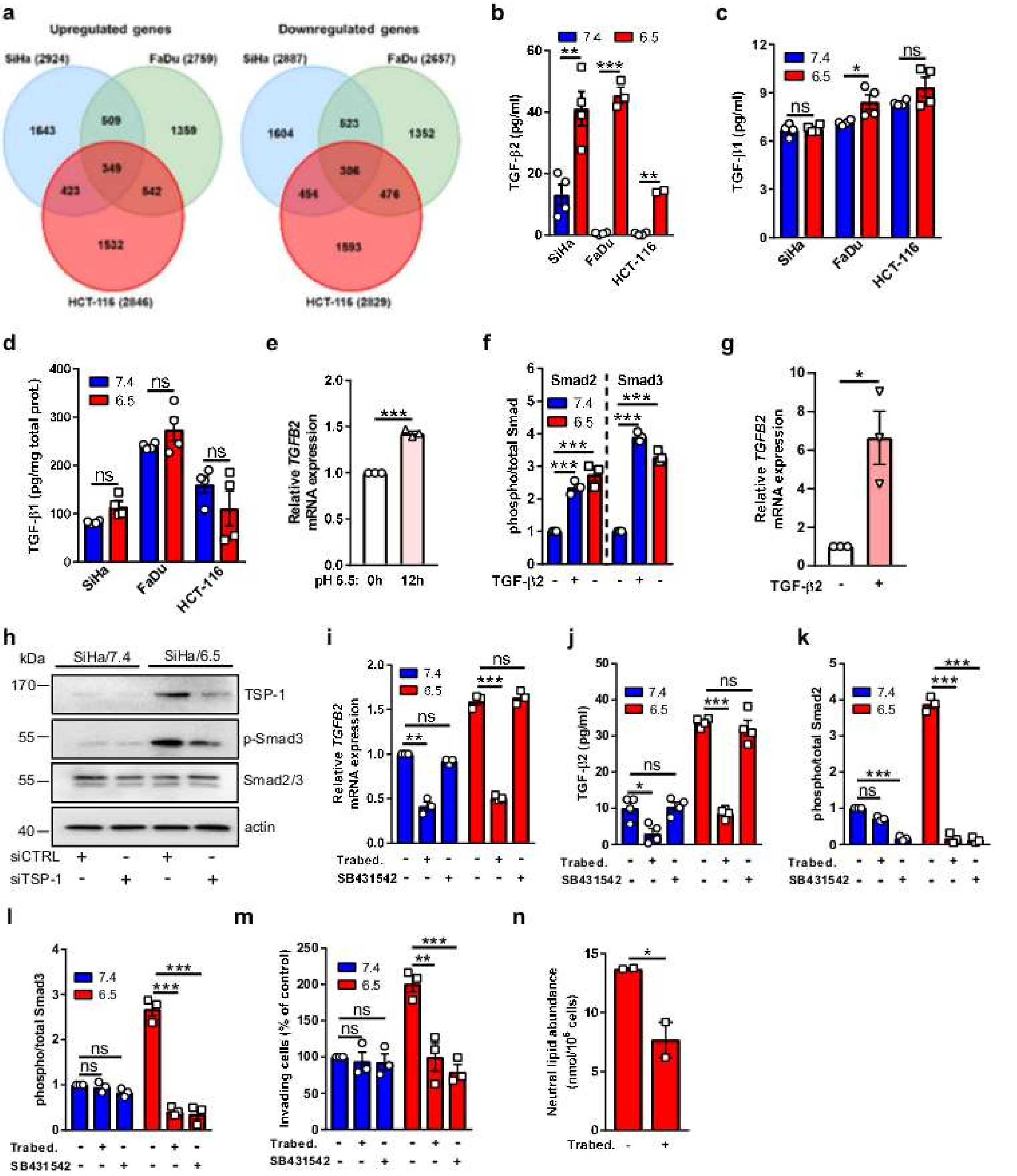

Supplementary Figure 3: TGF-β2 supports invasiveness and LD formation in acidosis-adapted cancer cells. (**a**) Venn diagrams showing overlap of up- and downregulated genes in pH 6.5-adapted SiHa, FaDu and HCT-116 cancer cell lines. Only the genes with a false discovery rate (FDR) < 0.01 and FPKM (fragments per kilobase of exon per million mapped reads) > 0.5 were considered for analysis. (**b**) Levels of active form of TGF-β2 in conditioned media (secreted) from native and acidosis-adapted tumor cells. (**c-d**) Levels of active form of TGF-β1 in conditioned media (secreted) (**c**) and in crude lysates (membrane-bound) (**d**) from

native and acidosis-adapted tumor cells. (**e**) mRNA expression for *TGFB2* in native SiHa cells exposed to acidic pH 6.5 for 12h. (**f**) Quantification of Smad2 (Ser465/467) and Smad3 (Ser423/425) phosphorylation levels in native and acidosis-adapted SiHa cells (with or without treatment with 4 ng/ml TGF-β2 for 6 hours). (**g**) mRNA expression for *TGFB2* in native SiHa cells following treatment with 4 ng/ml TGF-β2 for 24h. (**h**) Representative immunoblotting for phosphorylated and total forms of Smad3 (Ser423/425) and TSP-1 in native and acidosis-adapted SiHa cells following transfection of TSP1-targeting (or control) siRNA for 72h. (**i-l**) mRNA expression for *TGFB2* (**i**), levels of active form of TGF-β2 in conditioned media (secreted) (**j**), quantification of Smad2 (**k**) and Smad3 (**l**) phosphorylation levels in native and acidosis-adapted SiHa cells following treatment with 10 μM TGFβ2-specific antisense oligonucleotide Trabedersen for 7 days or 2 μM TGF-βRI inhibitor SB431542 for 24h. (**m**) Invasion capacity for native and acidosis-adapted HCT-116 cells following treatment as above. (**n**) Neutral lipid abundance in acidosis-adapted SiHa cells following treatment with 10 μM Trabedersen for 7 days. Data are represented as mean ± SEM of three independent experiments (with ≥6 technical replicates). Significance was determined by Student's t-test (**e, g, n**), or two-way ANOVA (**b-d, f, i-m**) with Bonferroni multiple-comparison analysis. *$p<0.05$; **$p<0.01$; ***$p<0.001$; ns, not significant. Source data are provided as a Source Data file.

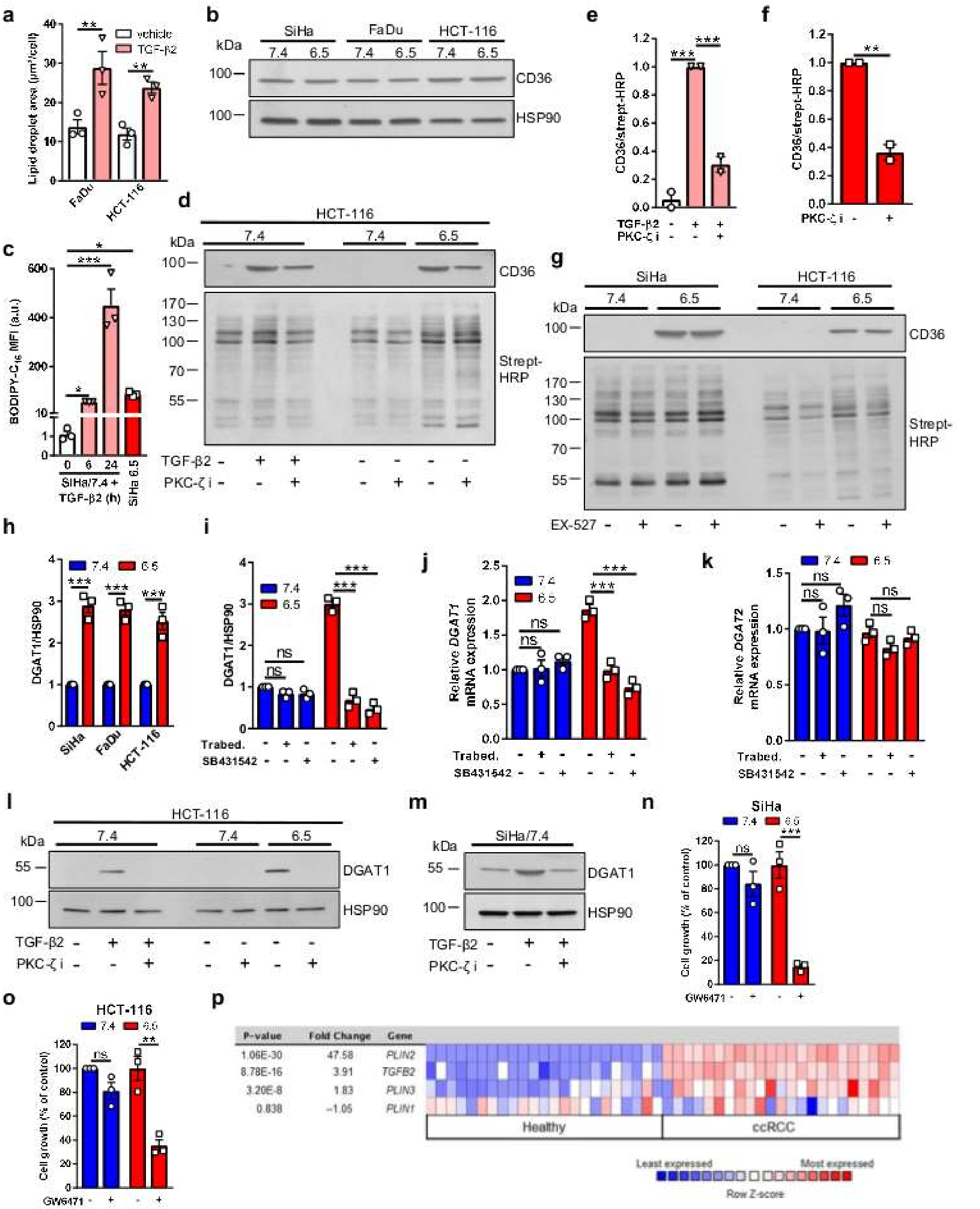

Supplementary Fig 4: TGF-β2 promotes FA uptake and TG accumulation into LD in a CD36 and DGAT1-dependent manner. (**a**) LD area in native FaDu and HCT-116 cells after treatment with 4 ng/ml TGF-β2 for 6h. (**b**) Representative immunoblotting for total CD36 in native and acidosis-adapted tumor cells. (**c**) Quantification for BODIPY FL C_{16} uptake in native and acidosis-adapted SiHa cells after treatment with 4 ng/ml TGF-β2 for 6h or 24h. (**d-f**)

Representative immunoblotting (**d**) and quantification (**e-f**) for cell surface-localized CD36 and total biotinylated proteins in native and acidosis-adapted HCT-116 cells following treatment with 4 ng/ml TGF-β2 and 10 μM PKC-ζ pseudo-substrate inhibitor for 24h. (**g**) Representative immunoblotting for cell surface-localized CD36 and total biotinylated proteins in native and acidosis-adapted tumor cells following treatment with 1 μM EX-527 (SIRT1 inhibitor) for 24h. (**h**) Quantification of DGAT1 protein levels in native and acidosis-adapted tumor cells. (**i-k**) Quantification of DGAT1 protein levels (**i**), mRNA expression for *DGAT1* (**j**) and *DGAT2* (**k**) in native and acidosis-adapted SiHa cells following treatment with 10 μM TGFβ2-specific antisense oligonucleotide Trabedersen for 7 days or 2 μM TGF-βRI inhibitor SB431542 for 24h. (**l-m**) Representative immunoblotting for DGAT1 in native and acidosis-adapted HCT-116 (**l**) or SiHa cells (**m**) following treatment with 4 ng/ml TGF-β2 and 10 μM PKC-ζ pseudo-substrate inhibitor for 24h. (**n-o**) Cell growth of native and acidosis-adapted SiHa (**n**) and HCT-116 cells (**o**) following treatment with 10 μM GW6471 (PPARα inhibitor) for 72h. (**p**) Co-expression analysis of *TGFB2*, *PLIN1*, *PLIN2* and *PLIN3* genes in human healthy and renal cancer (clear cell renal cell carcinoma; ccRCC) patient samples. Data are represented as mean ± SEM of three independent experiments (with ≥6 technical replicates). Significance was determined by Student's t-test (**f**), one-way ANOVA (**c, e**), or two-way ANOVA (**a, h-k, n-o**) with Bonferroni multiple-comparison analysis. *p<0.05; **p<0.01; ***p<0.001; ns, not significant. Source data are provided as a Source Data file.

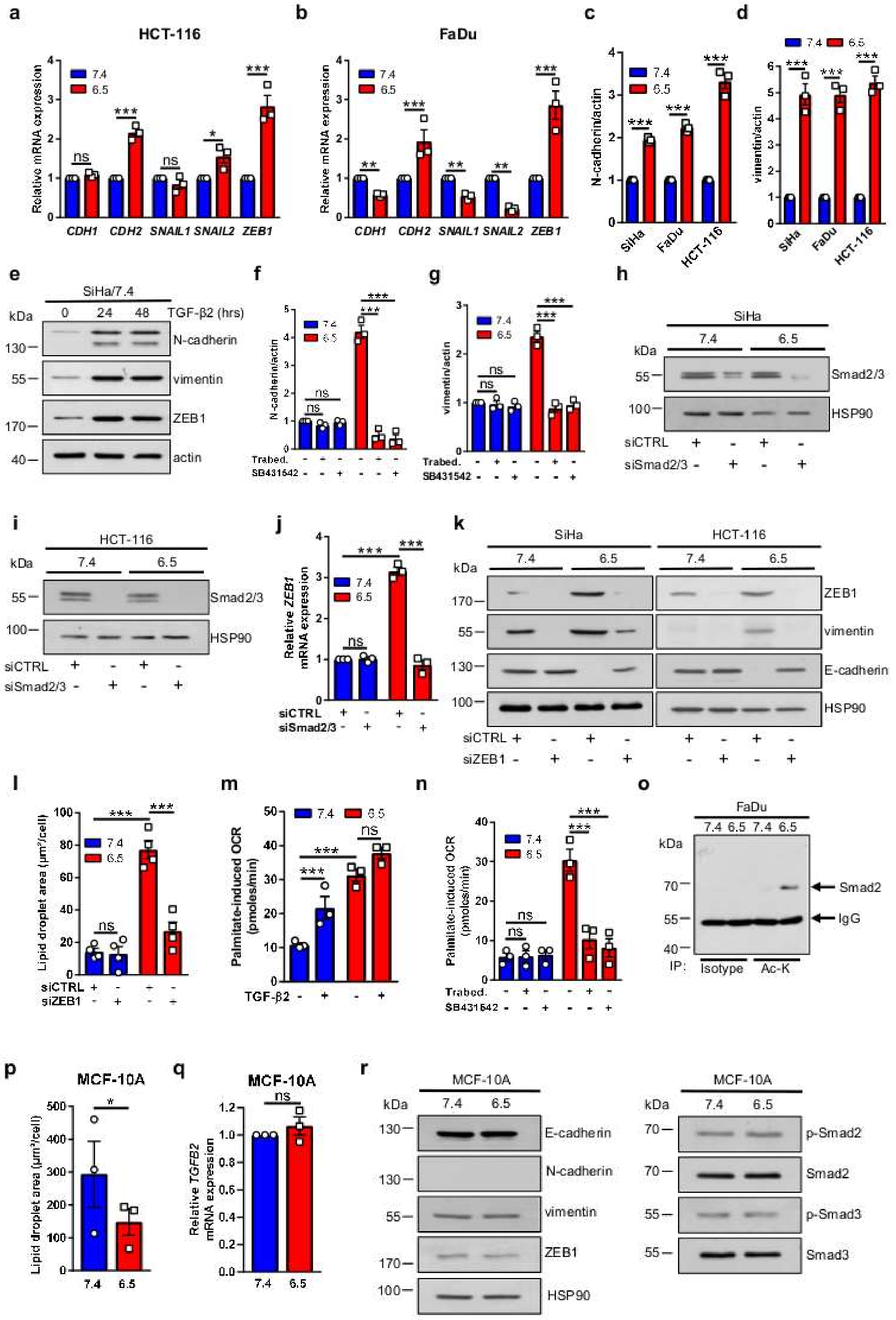
a
HCT-116
b
FaDu
c
d
e
SiHa/7.4
TGF-β2 (hrs)
N-cadherin
vimentin
ZEB1
actin
f
g
h
SiHa
Smad2/3
HSP90
siCTRL
siSmad2/3
i
HCT-116
j
k
SiHa
HCT-116
ZEB1
vimentin
E-cadherin
HSP90
siZEB1
l
m
n
o
FaDu
Smad2
IgG
Isotype
Ac-K
p
MCF-10A
q
MCF-10A
r
MCF-10A
E-cadherin
N-cadherin
vimentin
ZEB1
HSP90
MCF-10A
p-Smad2
Smad2
p-Smad3
Smad3

Supplementary Figure 5: EMT in acidosis-adapted cancer cells is driven by TGF-β2 and further enhanced by FAO-dependent Smad2 acetylation. (**a-b**) mRNA expression for some epithelial (*CDH1*) and mesenchymal (*CDH2*, *SNAIL1*, *SNAIL2* and *ZEB1*) genes in native and acidosis-adapted HCT-116 (**a**) and FaDu cells (**b**). (**c-d**) Quantification of N-cadherin (**c**) and vimentin (**d**) protein levels in native and acidosis-adapted tumor cells. (**e**) Representative immunoblotting for EMT-related protein markers in native SiHa cells following treatment with 4 ng/ml TGF-β2 for 24 and 48 hours. (**f-g**) Quantification of N-cadherin (**f**) and vimentin (**g**) protein levels in native and acidosis-adapted SiHa cells following treatment with 10 μM Trabedersen for 7 days or 2 μM SB431542 for 24h. (**h-j**) Representative immunoblotting for Smad2/3 in native and acidosis-adapted SiHa (**h**) and HCT-116 cells (**i**) and mRNA expression for *ZEB1* in native and acidosis-adapted HCT-116 cells (**j**) following transfection of Smad2/3-targeting (or control) siRNA for 72h. (**k-l**) Representative immunoblotting for EMT-related protein markers in native and acidosis-adapted tumor cells (**k**) and LD content in native and acidosis-adapted HCT-116 cells (**l**) following transfection with ZEB1-targeting (or control) siRNA for 72h. (**m-n**) Palmitate-dependent oxygen consumption rate in native and acidosis-adapted HCT-116 cells after treatment with 4 ng/ml TGF-β2 for 6h (**m**) or following treatment with 10 μM Trabedersen for 7 days or 2 μM SB431542 for 24h (**n**). (**o**) Representative immunoblotting for acetylated Smad2 in native and acidosis-adapted FaDu cancer cells. (**p-r**) LD content (**p**), mRNA expression for TGFB2 (**q**) and representative immunoblotting for EMT-related protein markers, phosphorylated and total forms of Smad2 (Ser465/467) and Smad3 (Ser423/425) (**r**) in non-tumorigenic MCF-10A mammary epithelial cells acutely exposed to acidic pH 6.5 for 48h (vs pH 7.4). Data are represented as mean ± SEM of three independent experiments (with ≥6 technical replicates). Significance was determined by Student's t-test (**p-q**), two-way ANOVA (**a-d, f-g, j, l-n**) with Bonferroni multiple-comparison analysis.*p<0.05; **p<0.01; ***p<0.001; ns, not significant. Source data are provided as a Source Data file.

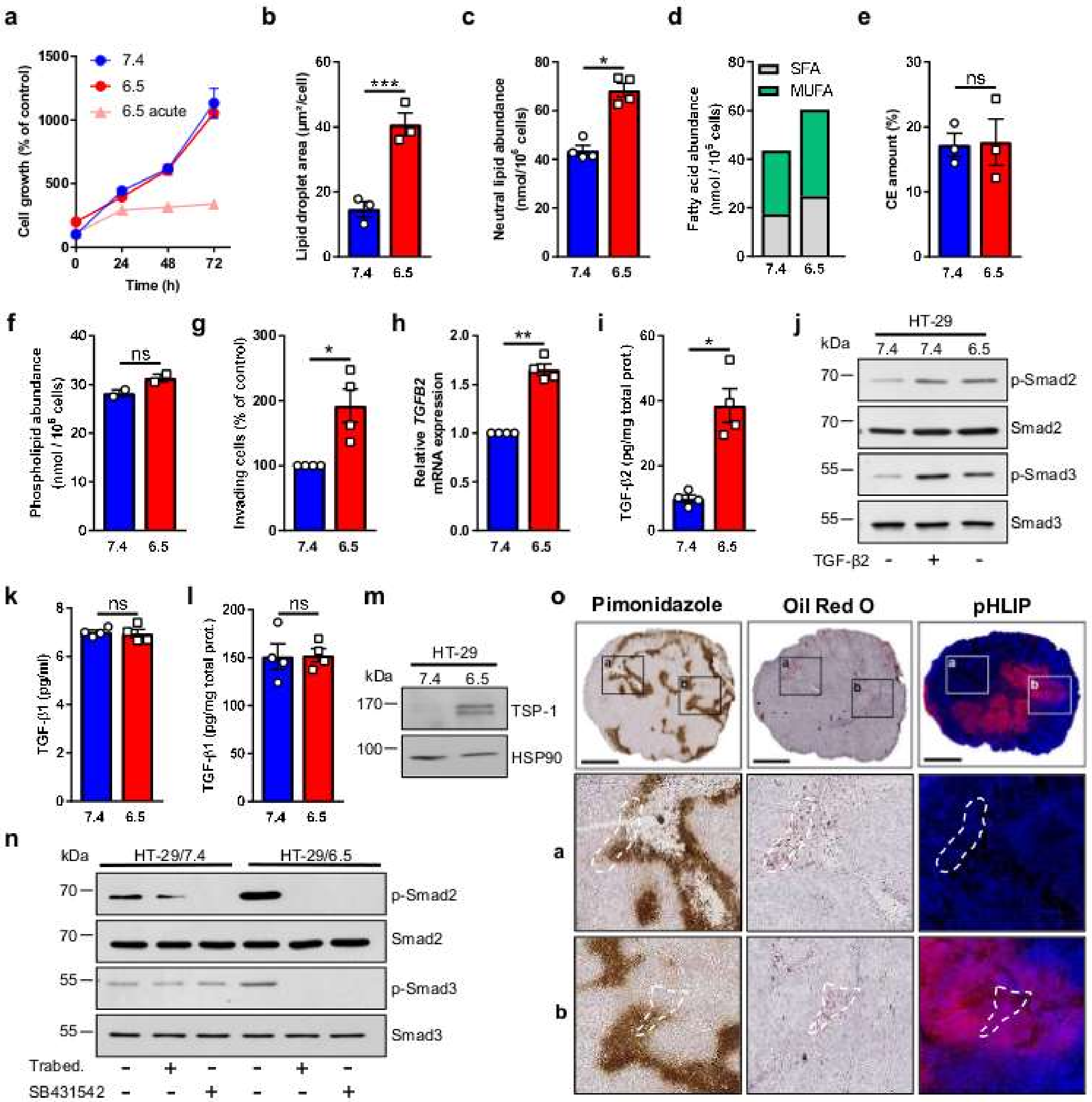

Supplementary Figure 6: EMT in acidosis-adapted cancer cells is driven by TGF-β2 and further enhanced by FAO-dependent Smad2 acetylation. (**a**) Time courses of the growth of native, chronically and acutely pH6.5-exposed HT-29 cancer cells. (**b-c**) LD content (**b**) and neutral lipid abundance (**c**) in native and acidosis-adapted HT-29 cells. (**d-e**) Abundance of SFA and MUFA (**d**) and cholesteryl esters (**e**) in the neutral lipid fraction of native and acidosis-adapted HT-29 cells. (**f**) Phospholipid abundance in native and acidosis-adapted HT-29 cells. (**g**) Invasion capacity of native and acidosis-adapted HT-29 cells. (**h-i**) mRNA expression (**h**) and levels of active form of TGF-β2 in crude lysates (membrane-bound) (**i**) from native and acidosis-adapted HT-29 cells. (**j**) Representative immunoblotting for phosphorylated and total forms of Smad2 (Ser465/467) and Smad3 (Ser423/425) in native and acidosis-adapted HT-29

cells (with or without treatment with 4 ng/ml TGF-β2 for 6 hours). (**k-l**) Levels of active form of TGF-β1 in conditioned media (secreted) (**k**) and in crude lysates (membrane-bound) (**l**) from native and acidosis-adapted HT-29 cells. (**m**) Representative immunoblotting for TSP-1 in native and acidosis-adapted HT-29 cells. (**n**) Representative immunoblotting for phosphorylated and total forms of Smad2 (Ser465/467) and Smad3 (Ser423/425) in native and acidosis-adapted HT-29 cells following treatment with 10 μM TGFβ2-specific antisense oligonucleotide Trabedersen for 7 days or 2 μM TGF-βRI inhibitor SB431542 for 24h. (**o**) Representative immunohistochemical and immunofluorescent pictures of hypoxic, LD-rich or acidic areas (using pimonidazole, Oil Red O and pHLIP markers, respectively) in subcutaneous HT-29 tumors. Source data are provided as a Source Data file.

Supplementary Table 1. Fatty acid abundance of the neutral lipid fraction in native SiHa cells treated or not for 6h with 4 ng/ml TGF-β2. Data indicated in the table are mean values of 2 biological replicates.

Saturated fatty acids (nmol/ 10^6 cells)		
	SiHa	
Fatty acid	CTRL	+ TGF-β2
C12:0	0.00	0.00
C14:0	0.30	0.07
C16:0	1.86	7.84
C18:0	1.24	5.65
C20:0	0.00	0.00
C22:0	0.00	0.00
C24:0	0.00	0.00
Total	3.40	13.56

Monounsaturated fatty acids (nmol/ 10^6 cells)		
	SiHa	
Fatty acid	CTRL	+ TGF-β2
C14:1cis9	0.00	0.00
C16:1cis9	0.10	0.57
C18:1cis9	1.10	2.30
C18:1cis11	0.31	0.63
C20:1c11	0.00	0.00
C22:1c13	0.00	0.00
C24:1c15	0.00	0.00
Total	1.51	3.50
TOTAL	4.91	17.05

Supplementary Table 2. Fatty acid abundance of the neutral lipid fraction in native and acidosis-adapted cancer cells. Data indicated in the table are mean values of 2 to 4 biological replicates.

**Saturated fatty acids (nmol/ 10^6 cells)**								
	**SiHa**		**FaDu**		**HCT-116**		**HT-29**	
**Fatty acid**	**7.4**	**6.5**	**7.4**	**6.5**	**7.4**	**6.5**	**7.4**	**6.5**
**C12:0**	0.00	0.10	0.43	0.49	0.13	0.16	0.09	0.12
**C14:0**	0.30	1.85	2.53	3.35	1.92	1.91	2.35	2.37
**C16:0**	2.97	7.52	6.52	12.28	5.04	10.88	11.28	16.39
**C18:0**	1.57	2.04	1.79	2.37	0.99	1.39	3.39	5.31
**C20:0**	0.00	0.07	0.02	0.00	0.00	0.08	0.18	0.34
**C22:0**	0.00	0.00	0.00	0.00	0.00	0.00	0.00	0.00
**C24:0**	0.00	0.02	0.11	0.13	0.00	0.12	0.15	0.27
**Total**	**4.84**	**11.61**	**11.41**	**18.62**	**8.08**	**14.54**	**17.45**	**24.79**
**Monounsaturated fatty acids (nmol/ 10^6 cells)**								
	**SiHa**		**FaDu**		**HCT-116**		**HT-29**	
**Fatty acid**	**7.4**	**6.5**	**7.4**	**6.5**	**7.4**	**6.5**	**7.4**	**6.5**
**C14:1cis9**	0.00	0.00	0.00	0.00	0.00	0.00	0.30	0.18
**C16:1cis9**	0.61	3.81	1.77	2.33	7.05	4.99	7.99	6.88
**C18:1cis9**	2.16	11.80	5.65	13.76	9.60	11.67	16.11	25.55
**C18:1cis11**	1.01	7.10	3.52	7.11	2.07	6.95	1.51	2.47
**C20:1c11**	0.07	0.58	0.30	1.23	0.52	0.70	0.14	0.39
**C22:1c13**	0.03	0.21	0.13	0.33	0.08	0.18	0.06	0.13
**C24:1c15**	0.00	0.24	0.20	0.53	0.14	0.17	0.01	0.07
**Total**	**3.87**	**23.74**	**11.57**	**25.28**	**19.47**	**24.66**	**26.13**	**35.67**
**TOTAL**	**8.71**	**35.35**	**22.98**	**43.90**	**27.55**	**39.20**	**43.58**	**60.46**

Supplementary Table 3. Sequences of gene-specific primers for qRT-PCR used in this study.

**Gene name**	**Forward primer (5' – 3')**	**Reverse primer (5' – 3')**
CDH1	TCACCACTGGGCTGGACCGA	TACAGCCTCCCACGCTGGGG
CDH2	CGAGCCGCCTGCGCTGCCAC	CGCTGCTCTCCGCTCCCCGC
DGAT1	CAACTACCGTGGCATCCTG	TTCTCCAGAAATAACCGGGC
DGAT2	CTGCACTGATTGCTGGCTCATCG	GAAAGTAGCGCCACACAGCCCAG
GTF2B	ACTACAGAGCCGGTGATATGAT	GTTGCTTTGTCATTGCTGAAAGT
PLIN1	CTCTCGATACACCGTGCAGA	TGGTCCTCATGATCCTCCTC
PLIN2	CTGCTCACGAGCTGCATCATC	TGTGAGATGGCAGAGAACGGT
PLIN3	GCTGGACAAGTTGGAGGAGA	CCGACACCTTAGACGACACA
PLIN4	TGCTGCAGAATGAGTTGGAG	GACCCAGGTCACCTAAACGA
PLIN5	AGCTTCCCTTTCTCCAGCAACCTT	AGTGATCCACCAGCTCCTCTGATT
SNAIL1	AATCCAGAGTTTACCTTCCAGCA	TCCCAGATGAGCATTGGCAG
SNAIL2	GAACTGGACACACATACAGTGAT	ACAGTGATGGGGCTGTATGC
TGFB2	TGTCCCTGCTGCACTTTTGTA	GGTGCCATCAATACCTGCAAATC
TGFBR1	GGCCAAATATCCCAAACAGA	TGATGCCTTCCTGTTGACTG
ZEB1	GCCCAAACTGCAAGAAACGC	GTCGCCCATTCACAGGTATCA
