# Supplementary material for: TGFβ2-induced formation of lipid droplets supports acidosis-driven EMT and the metastatic spreading of cancer cells
Source: Nat Commun. 2020 Jan 23;11:454. doi: 10.1038/s41467-019-14262-3 (PMC6978517; doi:10.1038/s41467-019-14262-3)
Supplement: Supplementary file 3 — Description of Additional Supplementary Files [file 41467_2019_14262_MOESM3_ESM.doc]

**Description of Additional Supplementary Files**

File Name: **Supplementary Data 1**

Description: **Lists of up- and downregulated genes in pH 6.5-adapted SiHa, FaDu and HCT-116 cancer cell lines used to draw the Venn diagrams in Figure S3A. Only the genes with a false discovery rate (FDR) < 0.01 and FPKM (fragments per kilobase of exon per million mapped reads) > 0.5 were considered for analysis. Full data from the RNA sequencing analysis reported in this paper were archived in GEO under the accession number GSE116035.**
