# Supplementary material for: TGFβ2-induced formation of lipid droplets supports acidosis-driven EMT and the metastatic spreading of cancer cells
Source: Nat Commun. 2020 Jan 23;11:454. doi: 10.1038/s41467-019-14262-3 (PMC6978517; doi:10.1038/s41467-019-14262-3)
Supplement: Supplementary file 5 — Reporting Summary [file 41467_2019_14262_MOESM5_ESM.pdf]

nature research

Corresponding author(s): Pr Olivier Feron
Dr Cyril Corbet

Last updated by author(s): Dec 14, 2019

Reporting Summary

Nature Research wishes to improve the reproducibility of the work that we publish. This form provides structure for consistency and transparency in reporting. For further information on Nature Research policies, see Authors & Referees and the Editorial Policy Checklist .

Statistics

For all statistical analyses, confirm that the following items are present in the figure legend, table legend, main text, or Methods section.

n/a	Confirmed	
☐	☒	The exact sample size (*n*) for each experimental group/condition, given as a discrete number and unit of measurement
☐	☒	A statement on whether measurements were taken from distinct samples or whether the same sample was measured repeatedly
☐	☒	The statistical test(s) used AND whether they are one- or two-sided<br>*Only common tests should be described solely by name; describe more complex techniques in the Methods section.*
☒	☐	A description of all covariates tested
☒	☐	A description of any assumptions or corrections, such as tests of normality and adjustment for multiple comparisons
☐	☒	A full description of the statistical parameters including central tendency (e.g. means) or other basic estimates (e.g. regression coefficient) AND variation (e.g. standard deviation) or associated estimates of uncertainty (e.g. confidence intervals)
☒	☐	For null hypothesis testing, the test statistic (e.g. *F*, *t*, *r*) with confidence intervals, effect sizes, degrees of freedom and *P* value noted<br>*Give P values as exact values whenever suitable.*
☒	☐	For Bayesian analysis, information on the choice of priors and Markov chain Monte Carlo settings
☒	☐	For hierarchical and complex designs, identification of the appropriate level for tests and full reporting of outcomes
☒	☐	Estimates of effect sizes (e.g. Cohen's *d*, Pearson's *r*), indicating how they were calculated

Our web collection on statistics for biologists contains articles on many of the points above.

Software and code

Policy information about availability of computer code

Data collection	No codes were used in data collection
Data analysis	No codes were used in data analysis

For manuscripts utilizing custom algorithms or software that are central to the research but not yet described in published literature, software must be made available to editors/reviewers. We strongly encourage code deposition in a community repository (e.g. GitHub). See the Nature Research guidelines for submitting code & software for further information.

Data

Policy information about availability of data

All manuscripts must include a data availability statement. This statement should provide the following information, where applicable:
- Accession codes, unique identifiers, or web links for publicly available datasets
- A list of figures that have associated raw data
- A description of any restrictions on data availability

The source data underlying all figures are provided as a Source Data file. The accession number for the RNA sequencing data reported in this paper is GEO: GSE116035 [https://www.ncbi.nlm.nih.gov/geo/query/acc.cgi?acc=GSE116035]. Any further information about resources and reagents should be directed to, and will be fulfilled by the corresponding authors upon reasonable request.

Field-specific reporting

Please select the one below that is the best fit for your research. If you are not sure, read the appropriate sections before making your selection.

Life sciences study design

All studies must disclose on these points even when the disclosure is negative.

Sample size	Sample sizes were chosen based on prior experience and similar reports published in Nature Comm; no statistical predermination of sample size was done.
Data exclusions	No data were excluded from the analyses.
Replication	Data presented in Figures were compiled from several biological replicates to ensure reproducibility.
Randomization	Mice were randomly allocated to experimental groups.
Blinding	The investigators were not blinded to group allocation but analyses were all validated by blinded collaborators.

Reporting for specific materials, systems and methods

We require information from authors about some types of materials, experimental systems and methods used in many studies. Here, indicate whether each material, system or method listed is relevant to your study. If you are not sure if a list item applies to your research, read the appropriate section before selecting a response.

Materials & experimental systems

n/a	Involved in the study	
☐	☒	Antibodies
☐	☒	Eukaryotic cell lines
☒	☐	Palaeontology
☐	☒	Animals and other organisms
☒	☐	Human research participants
☒	☐	Clinical data

Methods

n/a	Involved in the study	
☒	☐	ChIP-seq
☒	☐	Flow cytometry
☒	☐	MRI-based neuroimaging

Antibodies

Antibodies used	Primary antibodies against BiP (#3177), CPT1A (#12252), E-cadherin (#3195), N-cadherin (#13116), phospho-Smad2 (#3108); phospho-Smad3 (#9520), Smad2 (#5339), Smad3 (#9523), Smad2/3 (#8685), Snail (#3879), vimentin (#5741), ZEB1 (#3396), and ZO-1 (#8193), all from Cell Signaling Technology, were applied at 1:1,000 in 5% BSA overnight at 4°C. Anti-actin (#A5441; Sigma-Aldrich; 1:1,000), anti-CD36 (#100011; Cayman Chemical Company; 1:1,000), anti-DGAT1 (#ab54037; Abcam; 1:1,000), anti-HSP90 (#610419; BD Biosciences; 1:2,000), anti-perilipin 1 (#ab3526; Abcam; 1:1,000), anti-perilipin 2 (ab78920; Abcam; 1:1,000), anti-perilipin 5 (#PA1-46215; Thermo Fisher Scientific; 1:1,000), HRP-conjugated streptavidin (#SA10001; Thermo Fisher Scientific; 1:25,000) and anti-TSP1 (ab85762; Abcam; 1:1,000) antibodies were also applied in 5% BSA overnight at 4°C. In some experiments, cell lysates (1 mg) were first pre-cleared with 5 µg rabbit IgG (#02-6102; Thermo Fisher Scientific) and 30 µl Dynabeads Protein G (#10003D; Thermo Fisher Scientific) for 1h, at 4°C on an end-to-end roller, and then incubated with 5 µg anti-acetylated lysine (#9441; Cell Signaling Technology). For IHC, cells were incubated overnight at 4°C with anti-E-cadherin and anti-vimentin antibodies (#14472 and #5741, respectively; Cell Signaling Technology). Cells were then incubated with Alexa Fluor 488- and 568-conjugated anti-rabbit secondary antibodies (#A11034 and #A11036, respectively; Thermo Fisher Scientific) for 1h and nuclei were counterstained with DAPI.
Validation	Commercially available antibodies were validated by the vendors.

Eukaryotic cell lines

Policy information about cell lines

Cell line source(s)	Human cervix SiHa (#HTB-35), pharynx FaDu (#HTB-43) and colorectal HCT-116 (#CCL-247) and HT-29 (#HTB-38) cancer cell lines and the normal human breast epithelial cell line MCF-10A (#CRL-10317) were purchased from ATCC.
Authentication	Cells authenticated by ATCC were stored according to the supplier's instructions and used within 6 months after resuscitation of frozen aliquots.
Mycoplasma contamination	All cell lines were tested negative for mycoplasma contamination with the MycoAlert™ Mycoplasma Detection kit (#LT07-318; Lonza) before being used.
Commonly misidentified lines (See ICLAC register)	Non appl.

Animals and other organisms

Policy information about studies involving animals; ARRIVE guidelines recommended for reporting animal research

Laboratory animals	All experiments were performed with 7-week-old female Rj:NMRI-Foxn1nu/nu mice from Janvier Labs.
Wild animals	non appl.
Field-collected samples	non appl.
Ethics oversight	All experiments involving mouse xenograft and experimental metastasis models received the approval of the ethic committee from the Université Catholique de Louvain (approval ID 2016/UCL/MD018) and were carried out according to national care regulations.

Note that full information on the approval of the study protocol must also be provided in the manuscript.
